# The Standardization of Linear and Nonlinear Effects in Direct and Indirect Applications of Structural Equation Mixture Models for Normal and Nonnormal Data

**DOI:** 10.3389/fpsyg.2015.01813

**Published:** 2015-11-30

**Authors:** Holger Brandt, Nora Umbach, Augustin Kelava

**Affiliations:** Hector Research Institute of Education Sciences and Psychology, Eberhard Karls Universität TübingenTübingen, Germany

**Keywords:** interaction effect, quadratic effect, nonlinear effect, mixture model, nonnormality, standardization

## Abstract

The application of mixture models to flexibly estimate linear and nonlinear effects in the SEM framework has received increasing attention (e.g., Jedidi et al., 1997b; Bauer, 2005; Muthén and Asparouhov, 2009; Wall et al., 2012; Kelava and Brandt, 2014; Muthén and Asparouhov, 2014). The advantage of mixture models is that unobserved subgroups with class-specific relationships can be extracted (direct application), or that the mixtures can be used as a statistical tool to approximate nonnormal (latent) distributions (indirect application). Here, we provide a general standardization procedure for linear and nonlinear interaction and quadratic effects in mixture models. The procedure can also be applied to multiple group models or to single class models with nonlinear effects like LMS (Klein and Moosbrugger, 2000). We show that it is necessary to take nonnormality of the data into account for a correct standardization. We present an empirical example from education science applying the proposed procedure.

The estimation of nonlinear latent effects in the structural equation modeling (SEM) framework has received increasing attention over the last three decades. Several approaches for the analysis of nonlinear SEM have been published, which include among others the product indicator approaches (e.g., Kenny and Judd, 1984; Bollen, 1995; Jaccard and Wan, 1995; Jöreskog and Yang, 1996; Ping, 1995, 1996; Wall and Amemiya, 2001; Marsh et al., 2004; Little et al., 2006; Marsh et al., 2006; Kelava and Brandt, 2009), distribution analytic approaches (Klein and Moosbrugger, 2000; Klein and Muthén, 2007), moment based approaches (Wall and Amemiya, 2000, 2003; Mooijaart and Bentler, 2010), and Bayesian approaches (Arminger and Muthén, 1998; Lee, 2007; Kelava and Nagengast, 2012; Kelava and Brandt, 2014).

A typical structural model for a latent criterion η and two latent predictor variables (ξ_1_, ξ_2_) that includes one interaction effect and two quadratic effects is given by

(1) $$\begin{array}{l} \eta =\alpha +\gamma_{1}\xi_{1}+\gamma_{2}\xi_{2}+\gamma_{3}\xi_{1}\xi_{2}+\gamma_{4}\xi_{1}^{2}+\gamma_{5}\xi_{2}^{2}+\zeta \end{array}$$

where α is the latent intercept, the γs are the latent regression coefficients, and ζ is the latent residual.

In recent years, researchers have performed simulation studies to investigate the performance of several approaches to estimate latent nonlinear effects with structural equation models (e.g., Moulder and Algina, 2002; Wall and Amemiya, 2003; Marsh et al., 2004; Klein and Muthén, 2007; Cham et al., 2012; Brandt et al., 2014; Kelava et al., 2014). One of the main concerns in these studies was that nonlinear effects may be confounded with nonnormal distributions of the variables, which may lead to biased parameter or standard error estimates. For example, with the latent moderated structural equation modeling approach (LMS; Klein and Moosbrugger, 2000), it has been shown that it provides most efficient estimates under the condition of normally distributed predictors, but that this method produces spurious effects when data are nonnormally distributed (Kelava and Nagengast, 2012). Within the widely used class of the product indicator approaches, the unconstrained approach (Marsh et al., 2004, 2006; Kelava and Brandt, 2009) has become most popular, because it provides fairly robust parameter estimates (Marsh et al., 2004; Kelava and Nagengast, 2012) and in contrast to constrained approaches (Kenny and Judd, 1984; Jöreskog and Yang, 1996) it is comparatively easy to implement. Nevertheless, standard errors tend to be underestimated even in situations with normally distributed data, which leads to inflated Type I error rates (Kelava et al., 2011).

One conceptually different approach to modeling nonlinear effects is the use of semiparametric mixture models (SEMM; Arminger and Stein, 1997; Jedidi et al., 1997a,b; Dolan and van der Maas, 1998; Arminger et al., 1999; Muthén, 2001; Bauer and Curran, 2004; Bauer, 2005; Pek et al., 2009, 2011). Finite mixtures of linear SEM are used to approximate the unknown nonlinear relationship of the latent variables. While parametric approaches specify the functional form of their relationship a priori, the SEMM approach does not require assumptions about the functional form. In addition, the SEMM approach does not require the assumption of normally distributed latent variables and disturbances inherent in conventional SEM, but allows for flexible approximations of nonnormal distributions. Hence, the SEMM approach is a flexible tool for predicting the latent dependent variable if there is nonnormality and if obtaining a strict parametric representation of the functional relation does not have the highest priority (for a discussion see Bauer, 2005).

In general, mixture models can be applied with two different objectives. First, they can be used to identify unobserved groups within a heterogeneous population with linear and/or nonlinear group-specific relationships between the variables. In this kind of direct application, the mixture distributions are interpreted as representing distinct subpopulations. Second, mixture models can be used to approximate nonnormal distributions or nonlinear relationships *per se*, without assuming meaningful distinct subgroups in a population (McLachlan and Peel, 2000). These applications are then called indirect (see Bauer, 2005, for an application) and can be classified as a semiparametric approach to SEM. They have the advantage that they can be applied even when assumptions for traditional SEM are violated.

When a parametric representation of the nonlinear functional relation is of interest and nonnormality is given, an alternative is a recently proposed nonlinear structural equation mixture modeling approach (NSEMM; Kelava et al., 2014). With the NSEMM approach, nonnormality of the latent predictors can be approximated by applying a mixture of normal distributions (as an indirect application). Alternatively, the NSEMM approach can be used in a direct application, where heterogeneous subpopulations show different linear or nonlinear relations (e.g., interaction or quadratic effects) between latent variables.

## Standardization of SEM with parametric nonlinear effects

In general, as in manifest regression, it is common to use standardized regression coefficients in SEM in order to make effects comparable in their size and facilitate interpretation of results. For linear SEM, Bollen (1989, p. 165) defines a standardized coefficient $\gamma_{i}^{*}$ for a coefficient γ_*i*_ of a given predictor ξ_*i*_ (cp. Equation 1) as

(2) $$\begin{array}{l} \gamma_{i}^{*}:=\gamma_{i}\sqrt{\frac{\phi_{ii}}{\phi_{00}}} \end{array}$$

where ϕ_*ii*_ = *Var*(ξ_*i*_) and ϕ_00_ = *Var*(η). Standardized coefficients allow researchers to compare the effect sizes of predictors independent of the scaling of the predictor and the dependent variable. This argument also applies to interaction and quadratic effects. First, effect sizes are comparable across different studies. Second, simple slopes (see Aiken and West, 1991) based on standardized effects may give additional information about the importance of such nonlinear effects compared to simple slopes for unstandardized coefficients because they are also independent of the variables' scaling. Third, the size of linear effects depends on the means of the predictor variables when nonlinearity is present in the data. In order to make linear effects comparable in nonlinear models, researchers often center predictors before analyzing data; however, in some situations (e.g., in multiple group or latent class models; see below) this a priori centering is not possible. In these cases, an appropriate *post-hoc* standardization procedure is necessary that allows to compare linear effects in a meaningful way.

For mixture SEM and nonlinear SEM as introduced above, the standardization of parameter estimates needs some special considerations. For example, product terms of latent variables (e. g., ξ_1_ξ_2_ or $\xi_{1}^{2}$) have means and variances, which are not zero and one, respectively, even if the original variables were standard normally distributed (Bohrnstedt and Goldberger, 1969; Friedrich, 1982). Therefore, standardization procedures for linear SEM as produced by currently available software packages are not suitable for nonliner SEM.

For nonlinear SEM, Wen et al. (2010) addressed the problem of a correct standardization of the parameter estimates for SEM with single latent interaction effects. They showed that the standardized results (as given for linear SEM) provided by software programs are incorrect, because they refer to falsely standardized product variables. They provided formulas that allow one to transform an incorrectly standardized solution or an unstandardized solution to a correctly standardized solution. Following Wen et al.'s (2010) notation, the correctly standardized coefficients refer to an equation with correctly standardized variables (Friedrich, 1982; Wen et al., 2010), which is given as

(3) $$\begin{array}{l} \eta^{•}=\alpha^{•}+\gamma_{1}^{•}\xi_{1}^{•}+\gamma_{2}^{•}\xi_{2}^{•}+\gamma_{3}^{•}\xi_{1}^{•}\xi_{2}^{•}+\gamma_{4}^{•}{\left(\xi_{1}^{•}\right)}^{2}+\gamma_{5}^{•}{\left(\xi_{2}^{•}\right)}^{2}+\zeta^{•}. \end{array}$$

Note, that η^•^, $\xi_{1}^{•}$, and $\xi_{2}^{•}$ are standardized variables with zero means and unit variances. The product variables $\xi_{1}^{•}\xi_{2}^{•}$, ${\left(\xi_{1}^{•}\right)}^{2}$, and ${\left(\xi_{2}^{•}\right)}^{2}$ are products of standardized variables, but they are not standardized variables themselves (i.e., their means are not equal to zero and their variances are not equal to one, in general). A regression equation with standardized product variables of the form ${\left(\xi_{1}\xi_{2}\right)}^{*}=\left(\xi_{1}\xi_{2}-E\left[\xi_{1}\xi_{2}\right]\right)∕sd\left(\xi_{1}\xi_{2}\right)$, which have zero mean and unit variance would lead to an incorrectly standardized solution (as shown by Wen et al., 2010). As a consequence, $\gamma_{3}^{•}$, $\gamma_{4}^{•}$, and $\gamma_{5}^{•}$ in Equation (3) refer to the correctly standardized coefficients for the nonlinear model and $\gamma_{3}^{*}$, $\gamma_{4}^{*}$, and $\gamma_{5}^{*}$ refer to the standardized coefficients provided by standard statistic software that need additional corrections for interaction and quadratic effects.

The applicability of the formulas presented by Wen et al. (2010) suffers from three limitations. First, their standardization is limited to approaches that explicitly estimate (co-)variances of the latent product terms (e.g., the variance of ξ_1_ξ_2_ or the covariance between ξ_1_ξ_2_ and $\xi_{1}^{2}$). These (co-)variances can be retrieved from product indicator approaches or moment based approaches (e.g., Wall and Amemiya, 2003). They cannot be retrieved from distribution analytic approaches as LMS (Klein and Moosbrugger, 2000)—which is implemented as a standard estimator for nonlinear effects in Mplus (Muthén and Muthén, 1998–2012)—or QML (Klein and Muthén, 2007), because no (co-)variances of the latent product terms are estimated. As a consequence, neither a transformation from an incorrectly standardized solution provided by software programs (see Equation 12 in Wen et al., 2010) nor from an unstandardized solution (see Equation 11 and Supplementary Material (A.1) in Wen et al., 2010) is possible based on the formulas provided by Wen et al. (2010).

Second, the same is true for semiparametric approaches using mixtures of normal distributions to account for nonlinearity or nonnormality of the latent predictors (Jedidi et al., 1997b; Kelava and Nagengast, 2012; Kelava et al., 2014). In these approaches, the latent predictors' (nonnormal) distribution is approximated by a mixture of normal distributions with class-specific means and covariances. They have been shown to produce robust parameter estimates and are based on a latent class framework. The approaches do not provide estimates for the (co-)variances of the overall marginal distribution of the product terms and can be classified as distribution analytic approaches. Hence, the standardization procedure provided by Wen et al. (2010) can also not be used for this class of approaches.

Third, the procedure is restricted to models with centered latent predictor variables (see Wen et al., 2010, p. 4). This is problematic in situations where the means of the latent predictor variables have a substantive meaning. This can be the case in situations where multiple group models are estimated with known groups (Jöreskog, 1971; Sörbom, 1974) or latent classes where class membership is unknown a priori (Jedidi et al., 1997b). Then, typically the means are not restricted to be zero, but are estimated freely because they may contain information about the dissimilarities between the (a priori known or unknown) groups. If in fact nonlinearity is present in the data, the size of the linear effects depends on the means of the variables (e.g., Echambadi and Hess, 2007). Hence, the interpretation and the standardization of the linear effects need to be conducted with regard to the means of the variables involved in these situations.

## Scope of the article

In this article, we will provide the following extensions to previous work on the standardization of linear and nonlinear effects in SEM including latent mixtures: In the next section, we generalize the standardization procedure for nonlinear effects in SEM for (latent) class models with uncentered latent predictor variables. In situations with normally distributed data, this generalization allows for standardized parameter estimates of distribution analytic approaches, for example, LMS in Mplus, which do not provide explicit estimates of the (co-)variances of the latent product terms as compared to product indicator approaches. Furthermore, this generalization allows one to standardize (linear and nonlinear) group-specific regression coefficients for multiple group and latent class models in a direct application of mixture models.

Then, we derive formulas for the indirect application of semiparametric latent mixture SEM, which may or may not include nonlinear effects (Jedidi et al., 1997b; Bauer, 2005; Kelava and Nagengast, 2012; Kelava et al., 2014). The formulas we present are general in the sense that they can be applied to all mixture models including nonlinear effects, (e.g., to Growth Curve Mixture Models with interaction effects; Wen et al., 2014), or to multiple sample analyses with nonlinear effects when class membership is known a priori. The formulas provided can be used to standardize (non-)linear effects in situations with nonnormally distributed data (e.g., when applying the NSEMM approach). After the formal presentation, the proposed procedures are illustrated using data from education science.

## Standardization procedures

### Standardization of the direct application of a mixture model

For multiple class models with known or unknown class membership, the structural model specified in Equation (1) extends to

(4) $$\begin{array}{l} \eta_{g}=\alpha_{g}+\gamma_{1,g}\xi_{1,g}+\gamma_{2,g}\xi_{2,g}+\gamma_{3,g}\xi_{1,g}\xi_{2,g}\\ \;+\gamma_{4,g}\xi_{1,g}^{2}+\gamma_{5,g}\xi_{2,g}^{2}+\zeta_{g}, \end{array}$$

with *g* = 1, …, *G* (latent) classes. In this model, each regression coefficient may be class-specific. The latent residual is distributed as ζ_*g*_ ~ *N*(0, ψ_*g*_) and the latent predictors are distributed as ${\left(\xi_{1,g},\xi_{2,g}\right)}^{\prime }\sim N\left(\kappa_{g},\Phi_{g}\right)$ with class-specific parameters.

If the estimated parameters in the measurement model are allowed to differ across classes in addition to the proposed relaxation of the parameters in the structural model, the interpretation of the latent constructs is class-specific. To ensure that the latent constructs have the same meaning across classes, it is necessary to set the parameters of the measurement model equal across classes (intercepts, factor loadings and residual variances for strict measurement invariance, or intercepts and factor loadings for strong invariance, Meredith, 1993).

The standardization of the model given in Equation (4) is conducted within each class, such that the effect sizes can be calculated for each latent class separately. It is based on the correct within class standardization of η_*g*_, which is given by

(5) $$\begin{array}{l} \eta_{g}^{•}=\frac{\eta_{g}-E\left[\eta_{g}\right]}{sd\left(\eta_{g}\right)}=\frac{\eta_{g}-\kappa_{0,g}}{\sqrt{\phi_{00,g}}} \end{array}$$

with the class-specific (conditional) expectation κ_0, *g*_ and variance ϕ_00, *g*_ of η_*g*_. For the structural model given in Equation (4), the class-specific (conditional) expectation κ_0, *g*_ is given by

(6) $$\begin{array}{l} \kappa_{0,g}=\alpha_{g}+\gamma_{1,g}E\left[\xi_{1,g}\right]+\gamma_{2,g}E\left[\xi_{2,g}\right]+\gamma_{3,g}E\left[\xi_{1,g}\xi_{2,g}\right]\\ \;+\gamma_{4,g}E\left[\xi_{1,g}^{2}\right]+\gamma_{5,g}E\left[\xi_{2,g}^{2}\right]+E\left[\zeta_{g}\right]\\ \;=\alpha_{g}+\gamma_{1,g}\kappa_{1,g}+\gamma_{2,g}\kappa_{2,g}+\gamma_{3,g}\kappa_{3,g}+\gamma_{4,g}\kappa_{4,g}+\gamma_{5,g}\kappa_{5,g} \end{array}$$

with class-specific means κ_1, *g*_, κ_2, *g*_ of the latent predictors ξ_1, *g*_, ξ_2, *g*_. We assume that the latent residual term ζ_*g*_ has an expected value of zero within each class *g*. The expected values of the latent product terms are

(7) $$\begin{array}{l} \kappa_{3,g}=\kappa_{1,g}\kappa_{2,g}+\phi_{12,g}\;\kappa_{4,g}=\kappa_{1,g}^{2}+\phi_{11,g}\;\kappa_{5,g}=\kappa_{2,g}^{2}+\phi_{22,g} \end{array}$$

with class-specific variances ϕ_11, *g*_, ϕ_22, *g*_ of the predictors ξ_1, *g*_, ξ_2, *g*_, and covariance ϕ_12, *g*_. Note that these equations do not depend on any distributional assumption. The model implied variance ϕ_00, *g*_ of η_*g*_ is given in Supplementary Material A. Extending Equation (5) results in

(8) $$\begin{array}{l} \eta_{g}^{•}=\frac{\eta_{g}-\kappa_{0,g}}{\sqrt{\phi_{00,g}}}\;\\ =\frac{\alpha_{g}+\gamma_{1,g}\xi_{1,g}+\gamma_{2,g}\xi_{2,g}+\gamma_{3,g}\xi_{1,g}\xi_{2,g}+\gamma_{4,g}\xi_{1,g}^{2}+\gamma_{5,g}\xi_{2,g}^{2}+\zeta_{g}}{\sqrt{\phi_{00,g}}}\;\\ -\frac{\alpha_{g}+\gamma_{1,g}\kappa_{1,g}+\gamma_{2,g}\kappa_{2,g}+\gamma_{3,g}\kappa_{3,g}+\gamma_{4,g}\kappa_{4,g}+\gamma_{5,g}\kappa_{5,g}}{\sqrt{\phi_{00,g}}}\;\\ =\gamma_{1,g}\frac{\xi_{1,g}-\kappa_{1,g}}{\sqrt{\phi_{00,g}}}+\gamma_{2,g}\frac{\xi_{2,g}-\kappa_{2,g}}{\sqrt{\phi_{00,g}}}+\gamma_{3,g}\frac{\xi_{1,g}\xi_{2,g}-\kappa_{3,g}}{\sqrt{\phi_{00,g}}}\;\\ \;+\gamma_{4,g}\frac{\xi_{1,g}^{2}-\kappa_{4,g}}{\sqrt{\phi_{00,g}}}+\gamma_{5,g}\frac{\xi_{2,g}^{2}-\kappa_{5,g}}{\sqrt{\phi_{00,g}}}+\frac{\zeta_{g}}{\sqrt{\phi_{00,g}}}.\; \end{array}$$

Replacing the predictor variables ${\left(\xi_{1,g},\xi_{2,g}\right)}^{\prime }$ by their standardized versions $\xi_{1,g}=\xi_{1,g}^{•}\sqrt{\phi_{11,g}}+\kappa_{1,g}$ and $\xi_{2,g}=\xi_{2,g}^{•}\sqrt{\phi_{22,g}}+\kappa_{2,g}$ results in

(9) $$\begin{array}{l} \eta_{g}^{•}=\gamma_{1,g}\frac{\left(\xi_{1,g}^{•}\sqrt{\phi_{11,g}}+\kappa_{1,g}\right)-\kappa_{1,g}}{\sqrt{\phi_{00,g}}}\;\\ +\gamma_{2,g}\frac{\left(\xi_{2,g}^{•}\sqrt{\phi_{22,g}}+\kappa_{2,g}\right)-\kappa_{2,g}}{\sqrt{\phi_{00,g}}}\;\\ +\gamma_{3,g}\frac{\left(\xi_{1,g}^{•}\sqrt{\phi_{11,g}}+\kappa_{1,g}\right)\left(\xi_{2,g}^{•}\sqrt{\phi_{22,g}}+\kappa_{2,g}\right)-\kappa_{3,g}}{\sqrt{\phi_{00,g}}}\;\\ +\gamma_{4,g}\frac{{\left(\xi_{1,g}^{•}\sqrt{\phi_{11,g}}+\kappa_{1,g}\right)}^{2}-\kappa_{4,g}}{\sqrt{\phi_{00,g}}}\;\\ +\gamma_{5,g}\frac{{\left(\xi_{2,g}^{•}\sqrt{\phi_{22,g}}+\kappa_{2,g}\right)}^{2}-\kappa_{5,g}}{\sqrt{\phi_{00,g}}}+\frac{\zeta_{g}}{\sqrt{\phi_{00,g}}}.\; \end{array}$$

Standard algebra, some rearrangements, and substituting Equation (7) into Equation (9) leads to

(10) $$\begin{array}{l} \eta_{g}^{•}=\left(\gamma_{1,g}+\gamma_{3,g}\kappa_{2,g}+2\gamma_{5,g}\kappa_{1,g}\right)\sqrt{\frac{\phi_{11,g}}{\phi_{00,g}}}\xi_{1,g}^{•}\;\\ +\left(\gamma_{2,g}+\gamma_{3,g}\kappa_{1,g}+2\gamma_{5,g}\kappa_{2,g}\right)\sqrt{\frac{\phi_{22,g}}{\phi_{00,g}}}\xi_{2,g}^{•}\;\\ +\gamma_{3,g}\sqrt{\frac{\phi_{11,g}\phi_{22,g}}{\phi_{00,g}}}\xi_{1,g}^{•}\xi_{2,g}^{•}+\gamma_{4,g}\frac{\phi_{11,g}}{\sqrt{\phi_{00,g}}}{\left(\xi_{1,g}^{•}\right)}^{2}\;\\ +\gamma_{5,g}\frac{\phi_{22,g}}{\sqrt{\phi_{00,g}}}{\left(\xi_{2,g}^{•}\right)}^{2}\;\\ -\frac{\gamma_{3,g}\phi_{12,g}+\gamma_{4,g}\phi_{11,g}+\gamma_{5,g}\phi_{22,g}}{\sqrt{\phi_{00,g}}}+\frac{\zeta_{g}}{\sqrt{\phi_{00,g}}}.\; \end{array}$$

From this, the correctly standardized regression coefficients (see Equation 3) can be defined as:

(11) $$\begin{array}{l} \gamma_{1,g}^{•}:=\left(\gamma_{1,g}+\gamma_{3,g}\kappa_{2,g}+2\gamma_{4,g}\kappa_{1,g}\right)\sqrt{\frac{\phi_{11,g}}{\phi_{00,g}}}\; \end{array}$$

(12) $$\begin{array}{l} \gamma_{2,g}^{•}:=\left(\gamma_{2,g}+\gamma_{3,g}\kappa_{1,g}+2\gamma_{5,g}\kappa_{2,g}\right)\sqrt{\frac{\phi_{22,g}}{\phi_{00,g}}}\; \end{array}$$

(13) $$\begin{array}{l} \gamma_{3,g}^{•}:=\gamma_{3,g}\sqrt{\frac{\phi_{11,g}\phi_{22,g}}{\phi_{00,g}}}\; \end{array}$$

(14) $$\begin{array}{l} \gamma_{4,g}^{•}:=\gamma_{4,g}\frac{\phi_{11,g}}{\sqrt{\phi_{00,g}}}\; \end{array}$$

(15) $$\begin{array}{l} \gamma_{5,g}^{•}:=\gamma_{5,g}\frac{\phi_{22,g}}{\sqrt{\phi_{00,g}}}.\; \end{array}$$

An extension to more than two predictor variables and more than one dependent variable is straightforward.

Note that the formulations for the class-specific nonlinear effects in Equations (13–15) are similar to those given in Equation (10) and the discussion section by Wen et al. (2010). In contrast to the procedure suggested by Wen et al. (2010), we do not need an estimate for the (co-)variances of the product terms, but provide a formula for the model implied variance of η_*g*_ in Supplementary Material A. The procedure suggested here only requires information about the means and (co-)variances of the latent predictors ${\left(\xi_{1,g},\xi_{2,g}\right)}^{\prime }$, and the assumption of normally distributed predictors within each class. Setting *G* = 1 allows one to use the formulas for the LMS approach as well as all other approaches for the estimation of latent interaction and quadratic effects under the assumption of normally distributed predictors (for nonnormally distributed predictors, see the next section). The formulas are presented here for the transformation of the unstandardized coefficients because software programs (e.g., Mplus; Muthén and Muthén, 1998–2012) typically only provide unstandardized results for nonlinear models, especially when latent classes are extracted.

The standardized coefficients for the linear effects $\gamma_{1,g}^{•}$ and $\gamma_{2,g}^{•}$ depend on the class-specific means of the latent predictor variables (see also Moosbrugger et al., 1997). In general, linear effects are not independent of data transformations if nonlinear effects are present in the data. Hence, an interpretation of the linear effects as substantive effects should only be conducted when the predictors have a physically meaningful scale. Typically, these effects can only be interpreted meaningfully in combination with the nonlinear effects, in the sense of simple slopes (see Aiken and West, 1991). With the standardization proposed here, the linear effects are interpreted as the standardized linear relationship for subjects with an average value in the respective predictor variable. As a consequence, these effects can be interpreted in the same way as if the predictor variables had been centered prior to analysis. For the special case with zero means of the predictor variables, that is for κ_1, *g*_ = κ_2, *g*_ = 0, and *G* = 1, the formulas simplify to those provided by Wen et al. (2010), but note that centering the predictor variables is arbitrary and hence, the size of the (un-)standardized linear effects is arbitrary too.

If data are nonnormally distributed, it needs to be taken into account for the standardization because the variances and covariances between the latent product variables (and consequently the explained variance of the dependent variable) depend on the predictor variables' distribution. In the next section, we consider this by using a mixture model to approximate nonnormal distributions.

### Standardization for the indirect application of a mixture model

For the indirect application of the mixture model, the standardization is carried out across the mixture components. The restrictions imposed on the parameters constrain some or all parameters to be the same across classes except for the means and (co-)variances of the latent predictors. More specifically, for the NSEMM approach, all parameters except for the means and (co-)variances of the latent predictors are constrained across classes. This allows for a straightforward interpretation of the regression coefficients while the latent predictors' distribution can be approximated by the mixture model. In other indirect applications of SEMM approaches (e.g., Bauer, 2005) linear class-specific regression coefficients are used to approximate curvilinear relationships with unknown functional form. For such applications, a standardization cannot be conducted from a conceptual point because a standardized effect necessarily refers to a specific parametric effect with an a priori defined functional relationship between the variables.

In the NSEMM approach, the measurement model is restricted to be equal across classes and the structural model from Equation (4) is constrained such that

(16) $$\begin{array}{l} \eta_{g}=\alpha +\gamma_{1}\xi_{1,g}+\gamma_{2}\xi_{2,g}+\gamma_{3}\xi_{1,g}\xi_{2,g}+\gamma_{4}\xi_{1,g}^{2}+\gamma_{5}\xi_{2,g}^{2}+\zeta_{g}\; \end{array}$$

with the latent residual distributed as ζ_*g*_ ~ *N*(0, ψ). The latent predictors are distributed as $\xi_{g}={\left(\xi_{1,g},\xi_{2,g}\right)}^{\prime }\sim N\left(\kappa_{g},\Phi_{g}\right)$ within each class. The overall distribution of **ξ**_*g*_ is a nonnormal mixture distribution (see Haas et al., 2009) with mean vector

(17) $$\begin{array}{l} \kappa =\overset{G}{\underset{g=1}{\sum }}\pi_{g}\kappa_{g}\; \end{array}$$

and covariance matrix

(18) $$\begin{array}{l} \Phi =\overset{G}{\underset{g=1}{\sum }}\pi_{g}\left(\Phi_{g}+\kappa_{g}\kappa_{g}^{\prime }\right)-\kappa \kappa^{\prime }.\; \end{array}$$

The standardized coefficients are defined analogously to Equations (11–15) without the class-specificity:

(19) $$\begin{array}{l} \gamma_{1}^{•}:=\left(\gamma_{1}+\gamma_{3}\kappa_{2}+2\gamma_{4}\kappa_{1}\right)\sqrt{\frac{\phi_{11}}{\phi_{00}}}\; \end{array}$$

(20) $$\begin{array}{l} \gamma_{2}^{•}:=\left(\gamma_{2}+\gamma_{3}\kappa_{1}+2\gamma_{5}\kappa_{2}\right)\sqrt{\frac{\phi_{22}}{\phi_{00}}}\; \end{array}$$

(21) $$\begin{array}{l} \gamma_{3}^{•}:=\gamma_{3}\sqrt{\frac{\phi_{11}\phi_{22}}{\phi_{00}}}\; \end{array}$$

(22) $$\begin{array}{l} \gamma_{4}^{•}:=\gamma_{4}\frac{\phi_{11}}{\sqrt{\phi_{00}}}\; \end{array}$$

(23) $$\begin{array}{l} \gamma_{5}^{•}:=\gamma_{5}\frac{\phi_{22}}{\sqrt{\phi_{00}}}.\; \end{array}$$

The means and the (co-)variances of the latent predictors are the elements of the mean vector and the covariance matrix in Equations (17) and (18) (see Supplementary Material A for the general formulas for the moments of mixture variables). An extension to more than two predictor variables and more than one dependent variable again is straightforward.

The model implied variance ϕ_00_ of η_*g*_ again is given in Supplementary Material A. Note that for the derivation of this variance we do not assume that the predictor variables are normally distributed. As a consequence, third and fourth moments of the variables need to be taken into account for the calculation of the model implied variance of η_*g*_. Here, we suggest to use the moments of the mixture distributions that approximate the nonnormality of the latent predictors as an estimate for these higher order moments. The derivation of the model implied variance of the latent dependent variable shows that it is essential to take nonnormality of the predictor variables into account when standardizing linear and nonlinear effects.

### Standardization in multiple group models with observed groups

In applications of multiple group models, class-specific regression coefficients are not always standardized using the within-class means and variances. However, they can be standardized using the pooled means and variances across groups, or they can be standardized using those of a reference group. If researchers want to use pooled parameters in order to have a common reference system for different subgroups, the means and variances of an indirect application of a mixture model (given in Equations (19–23)) can be used instead of those for a within group standardization based on the formulas given in Equations (11–15).

The decision whether a standardization should be conducted using the within variances or the pooled variances should be based on the comparisons that the user wants to make. If different groups within a study are compared to each other (e.g., female and male students) then a pooled variance is meaningful because differences across groups (e.g., means) are maintained and represented in the standardized results. The frame of reference is defined by the pooled means and variances. As a consequence, standardized coefficients refer to a common metric in the study.

If estimates are compared across studies for specific subgroups (e.g., female students across different studies in a meta analysis) then a within standardization is meaningful because the frame of reference is then defined by group-specific parameters. The pooled variances could lead to wrong interpretations because they are influenced by the group proportions in each study; if these proportions are different across studies (e.g., 10% females in one study and 80% in another study) a comparison of standardized effects based on the pooled variance can be misleading (this will be the case when, e.g., the variances of male and female students are clearly different because the weighted sums of the means and variances depend on the group proportions).

In Supplementary Material B, we illustrate the two standardization procedures with a fictitious data set on shoe size and body height.

## Empirical example

In this section, we illustrate the standardization procedure for direct and indirect applications of nonlinear structural equation mixture models with an example based on data from the Program for International Student Assessment 2009 (PISA; Organisation for Economic Co-Operation and Development, 2010), which is publicly available under http://pisa2009.acer.edu.au/downloads.php. The sample was an Australian subsample of *N* = 1092 students who took part in a reading test. Students' attitude toward reading (*Att*) and their reported online activities (*Online*; i.e., read emails or chat online) were selected as predictors of reading skills (*Read*). For simplicity, we neglected the clustered data structure due to the schools that had only a minor impact on the reading skills outcome (*ICC* = 0.15)^1^.

In addition to the linear effects, we also tested the nonlinear structure between the variables and included one interaction and two quadratic effects in each model to increase the predictive power of the model. We assumed that students with a positive attitude toward reading (i.e., students with high values in *Att*) benefit more in terms of their reading skills from engaging in online reading activities. We operationalized this hypothesis with an interaction between *Att* and *Online*. Furthermore, we formulated quadratic effects for both latent predictor variables based on recommendations by Ganzach (1997) and Klein et al. (2009) because quadratic effects should be included into an interaction model in order to avoid spurious interaction effects. We also based this decision on the assumption that a positive reading attitude and online activities do not linearly increase reading skills; instead, we assumed that there may be a saturation effect which was operationalized by a negative quadratic effect.

In addition to the structural model, we included different assumptions with regard to observed or unobserved groups. First, we were interested if different latent classes with distinct linear and nonlinear effects could be extracted from the data. This may offer insights about how reading skills may be class-specific and therefore if different subpopulations need to be assumed. This model is a direct application of a mixture model [Model (a)]. Second, in Model (b), we tested whether the latent predictors were nonnormally distributed, while we assumed a global relationship between the variables for all students. This model is an indirect application of a mixture model and allows for an unbiased estimation of the latent nonlinear effects if nonnormality is present in the data. Third, we tested whether gender effects could be identified in the data [Model (c)]. Previous research has indicated that attitudes toward reading as well as reading skills differ for girls and for boys (Schiefele, 2009; Wigfield and Cambria, 2010). Here, we also tested whether the relationships between these variables differ across girls and boys (for the gender specific relationship between interest and achievement, see Schiefele et al., 1992).

### Model formulation

#### Measurement model

Between 7 and 11 items were aggregated into three indicators for each latent variable (i.e., item parcels) for didactic purposes^2^. The measurement models were given by

(24) $$\begin{array}{l} \text{y}=\tau +\Lambda {\left(Att,Online,Read\right)}^{\prime }+\epsilon ,\; \end{array}$$

with an intercept vector **τ**, a factor loading matrix **Λ** and a residual vector **ϵ**. The factor loading matrix **Λ** was formulated with a simple structure (i.e., each item loaded only on one latent variable). The residual variables **ϵ** were assumed to be mutually uncorrelated and normally distributed with zero mean vector and (diagonal) covariance matrix **Θ**. For the identification of the model, the intercept and the factor loading of the first indicator of each latent variable were fixed to zero and one, respectively.

#### Structural models

We specified three different types of structural models: (a) a direct application of a mixture model with varying regression coefficients across classes and (b) an indirect application with fixed regression coefficients across classes. Each of these models (a) and (b) were specified with two or three latent classes. Finally, Model (c) was a multiple group model with gender as a grouping variable and varying regression coefficients across groups. For comparison, a single class model was analyzed, too.

More specifically, Model (a) was specified as

(25) $$\begin{array}{l} Read_{g}=\alpha_{g}+\gamma_{1,g}Att_{g}+\gamma_{2,g}Online_{g}+\gamma_{3,g}Att_{g}\cdot Online_{g}\;\\ \;+\gamma_{4,g}Att_{g}^{2}+\gamma_{5,g}Online_{g}^{2}+\zeta_{g},\; \end{array}$$

with a normally distributed residual ζ_*g*_ ~ *N*(0, ψ_*g*_) and predictors ${\left(Att_{g},Online_{g}\right)}^{\prime }\sim N\left(\kappa_{g},\Phi_{g}\right)$ within each latent class *g*.

Model (b) was specified for the indirect application as

(26) $$\begin{array}{l} Read_{g}=\alpha +\gamma_{1}Att_{g}+\gamma_{2}Online_{g}+\gamma_{3}Att_{g}\cdot Online_{g}\;\\ \;+\gamma_{4}Att_{g}^{2}+\gamma_{5}Online_{g}^{2}+\zeta_{g},\; \end{array}$$

with a normally distributed residual ζ_*g*_ ~ *N*(0, ψ) and predictors ${\left(Att_{g},Online_{g}\right)}^{\prime }\sim N\left(\kappa_{g},\Phi_{g}\right)$ within each latent class *g*. The latent classes in this model were used to approximate the potentially nonnormal distribution of the latent variables *Att* and *Online*.

Model (c) was specified as a multiple group model with gender as a grouping variable, such that reading skills were predicted separately for male and female students, whereby the measurement model was invariant across groups. The structural model was formulated according to Equation (25).

All models were estimated in Mplus (Muthén and Muthén, 1998–2012).

### Results

#### Model selection

The model fit indices for the three models with different class solutions are presented in Table 1. For the direct application, the two class solution was selected as the final model because the BIC value was smaller compared to the three class solution. For the indirect application, the two class solution provided the best fit. The larger fit indices for the single class solution indicated the necessity to account for nonnormality in the data when analyzing nonlinear effects (for details see Kelava et al., 2014).

**Table 1:** **Model fit indices for the three models**.

**Number of classes**	**AIC**	**BIC**	**Adjusted BIC**
**DIRECT APPLICATION [MODEL (A)]**			
2	9,899	10,133	9,984
3	9,889	10,188	9,998
**INDIRECT APPLICATION [MODEL (B)]**			
1	10,094	10,264	10,156
2	10,046	10,246	10,119
3	10,118	10,348	10,202
**MULTIPLE GROUP MODEL [MODEL (C)]**			
2	11,516	11,751	11,601

**Figure 1:**
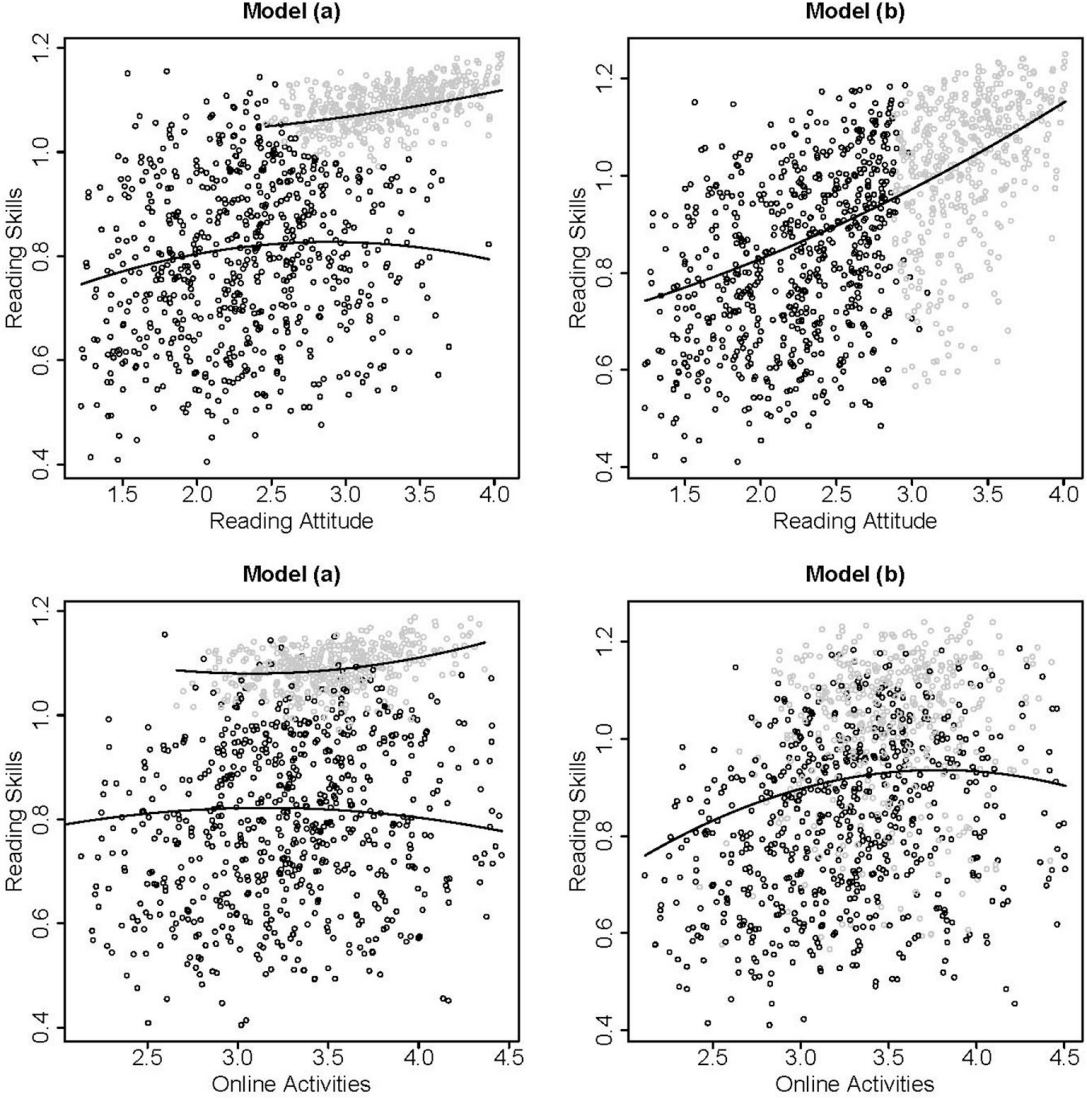
**Scatter plots of the estimated factor scores based on the two class solutions of Model (a) (left panels) and Model (b) (right panels) for reading attitude and reading skills (upper panels) and online activities and reading skills (lower panels)**. The model based relationships between the variables are indicated with solid lines, class membership is indicated by black or gray dots.

In this analysis, we did not evaluate whether the direct or the indirect application [Model (a) or Model (b)] was more appropriate because the application of either model should be based on theory. While Model (a) aims to extract subgroups with homogeneous patterns, Model (b) fits a model for all subjects and only accounts for nonnormality by the latent class model. Thus, the latent classes are not interpreted as subgroups. Figure 1 illustrates the differences between Model (a) and (b). The subgroups in Model (a) were distinguished especially by their reading skills and attitudes. The second class consisted of a very homogeneous group of subjects with high reading skills and attitudes (see estimates in **Table 3**, *C* = 2). In Model (b) differences in the subgroups were related only to high or low reading attitudes. This implies that the better model fit for the two class solution compared to the single class solution was caused by the nonnormality of the reading attitudes in this example.

#### Parameter estimates for model (a)

The results of the two class solution of Model (a), which is the direct application of a mixture model with class-specific (non)-linear effects, are presented in Table 2. None of the nonlinear effects were significant, each of these effects were close to zero and had a standardized absolute effect size between 0.029 and 0.104. The only significant linear effects were γ_1, 1_ and γ_2, 1_ in class 1; however, the effect sizes of the linear effects in the two groups were different with ${\hat{\gamma }}_{1,1}^{•}=0.122$ and ${\hat{\gamma }}_{2,1}^{•}=0.116$ in class 1, and ${\hat{\gamma }}_{1,2}^{•}=0.384$ and ${\hat{\gamma }}_{2,2}^{•}=0.229$ in class 2 (although this difference was not significant with *p* = 0.202 and *p* = 0.768, respectively). This indicated nonlinearity in the data, which was accounted for by the two classes (see details for modeling semiparametric curvilinear relationships with class-specific linear effects in Bauer, 2005). The explained variance was smaller in class 1 with 6% compared to class 2 with 24% (1−0.942 = 0.058 in class 1 and 1−0.756 = 0.244 in class 2).

**Table 2:** **Unstandardized and standardized parameter estimates, standard errors, ***t***- and ***p***-values for the direct application of a nonlinear model with two latent classes**.

	**$\hat{\theta }$**	***SE***	**${\hat{\theta }}^{•}$**	***t***	***p***
*P*(*C* = 1)	0.626				
*P*(*C* = 2)	0.374				
***C* = 1**					
γ_1, 1_	0.309	0.144	0.122	2.152	0.031
γ_2, 1_	0.305	0.148	0.116	2.065	0.039
γ_3, 1_	−0.042	0.030	−0.079	−1.423	0.155
γ_4, 1_	−0.029	0.027	−0.058	−1.094	0.274
γ_5, 1_	−0.026	0.024	−0.046	−1.066	0.287
κ_1, 1_	2.341	0.037	0.000	62.662	< 0.001
κ_2, 1_	3.272	0.029	0.000	110.979	< 0.001
α_1_	−0.147	0.294	0.000	−0.500	0.617
ϕ_11, 1_	0.344	0.026	1.000	13.202	< 0.001
ϕ_21, 1_	0.063	0.016	0.195	3.843	< 0.001
ϕ_22, 1_	0.302	0.044	1.000	6.805	< 0.001
ψ_1_	0.028	0.003	0.942	9.845	< 0.001
***C* = 2**					
γ_1, 2_	−0.045	0.178	0.384	−0.254	0.799
γ_2, 2_	−0.252	0.288	0.229	−0.875	0.381
γ_3, 2_	0.009	0.034	0.029	0.268	0.789
γ_4, 2_	0.009	0.024	0.033	0.395	0.693
γ_5, 2_	0.036	0.040	0.104	0.898	0.369
κ_1, 2_	3.276	0.043	0.000	76.413	< 0.001
κ_2, 2_	3.514	0.033	0.000	104.996	< 0.001
α_2_	1.471	0.578	0.000	2.544	0.011
ϕ_11, 2_	0.188	0.020	1.000	9.230	< 0.001
ϕ_21, 2_	0.016	0.015	0.096	1.057	0.290
ϕ_22, 2_	0.149	0.022	1.000	6.643	< 0.001
ψ_2_	0.002	0.001	0.756	2.273	0.023

*$\hat{\theta }$, unstandardized parameter estimates; SE, estimated standard errors; ${\hat{\theta }}^{•}$, standardized parameter estimates; t, t-values based on standard errors; p, p-values for the t-values*.

#### Parameter estimates for model (b)

**Figure 2:**
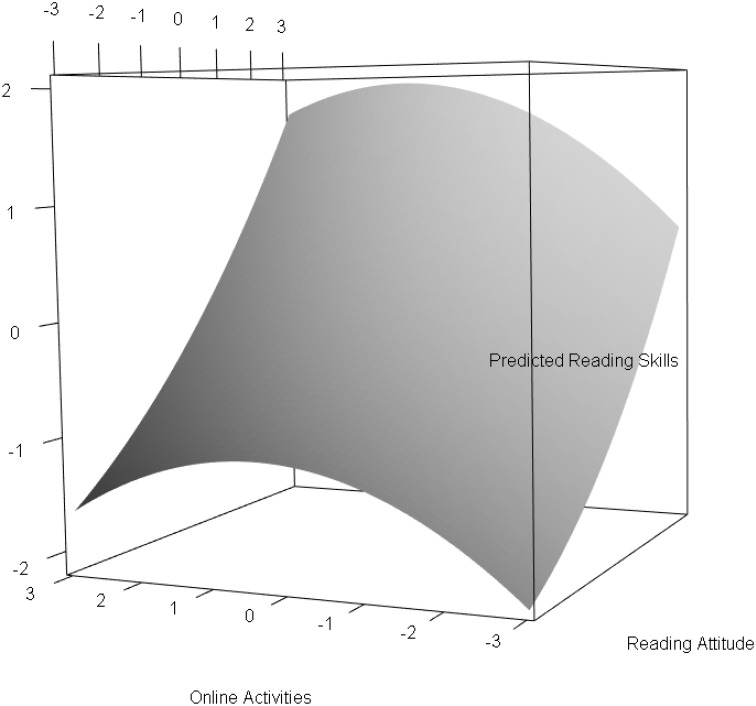
**Standardized multivariate relationship between reading attitude, online activities and reading skills**.

The results for the two class solution of Model (b), which is the indirect application of a mixture model with (non)-linear effects, are presented in Table 3. In this model, the online activities had a significant negative quadratic effect on reading skills with an effect size of ${\hat{\gamma }}_{5}^{•}=-0.085$. The standardized linear effect for reading attitude was strong with ${\hat{\gamma }}_{1}^{•}=0.514$. This effect can be interpreted as the effect for subjects with an average level of reading attitude. The standardized multivariate relationship between reading attitude, online activities, and reading skills are illustrated in Figure 2. The nonlinear relationship between online activities and reading skills modeled a saturation effect; that is, for subjects with standardized online activities between –3 and 0, a positive relation with reading skills was observed. For subjects with standardized online activities between 0 and 3, the reading skills only changed marginally. The explained variance in the model was 35% (1 − 0.647).

**Table 3:** **Unstandardized and standardized parameter estimates, standard errors, ***t***- and ***p***-values for the indirect application of a nonlinear model with two latent classes**.

	**$\hat{\theta }$**	***SE***	**${\hat{\theta }}^{•}$**	***t***	***p***
*P*(*C* = 1)	0.606				
*P*(*C* = 2)	0.394				
γ_1_	0.025	0.078	0.514	0.324	0.746
γ_2_	0.450	0.145	0.137	3.107	0.002
γ_3_	0.010	0.023	0.018	0.413	0.679
γ_4_	0.017	0.012	0.041	1.344	0.179
γ_5_	−0.063	0.025	−0.085	−2.510	0.012
κ_1_	2.691	0.023	0.000	117.042	< 0.001
κ_2_	3.362	0.021	0.000	161.332	< 0.001
α	−0.157	0.243	0.000	−0.647	0.517
ϕ_11_	0.488	0.023	1.000	21.682	< 0.001
ϕ_21_	0.102	0.013	0.277	7.856	< 0.001
ϕ_22_	0.274	0.046	1.000	6.132	< 0.001
ψ	0.027	0.002	0.649	11.205	< 0.001
***C* = 1**					
κ_1, 1_	2.273	0.228	0.000	9.982	< 0.001
κ_2, 1_	3.274	0.091	0.000	35.906	< 0.001
ϕ_11, 1_	0.272	0.097	1.000	2.819	0.005
ϕ_21, 1_	0.063	0.050	0.214	1.270	0.204
ϕ_22, 1_	0.318	0.049	1.000	6.445	< 0.001
***C* = 2**					
κ_1, 2_	3.333	0.207	0.000	16.119	< 0.001
κ_2, 2_	3.498	0.055	0.000	63.662	< 0.001
ϕ_11, 2_	0.140	0.068	1.000	2.053	0.040
ϕ_21, 2_	0.017	0.023	0.108	0.721	0.471
ϕ_22, 2_	0.177	0.046	1.000	3.834	< 0.001

*$\hat{\theta }$, unstandardized parameter estimates; SE, estimated standard errors; ${\hat{\theta }}^{•}$, standardized parameter estimates; t, t-values based on standard errors; p, p-values for the t-values*.

**Figure 3:**
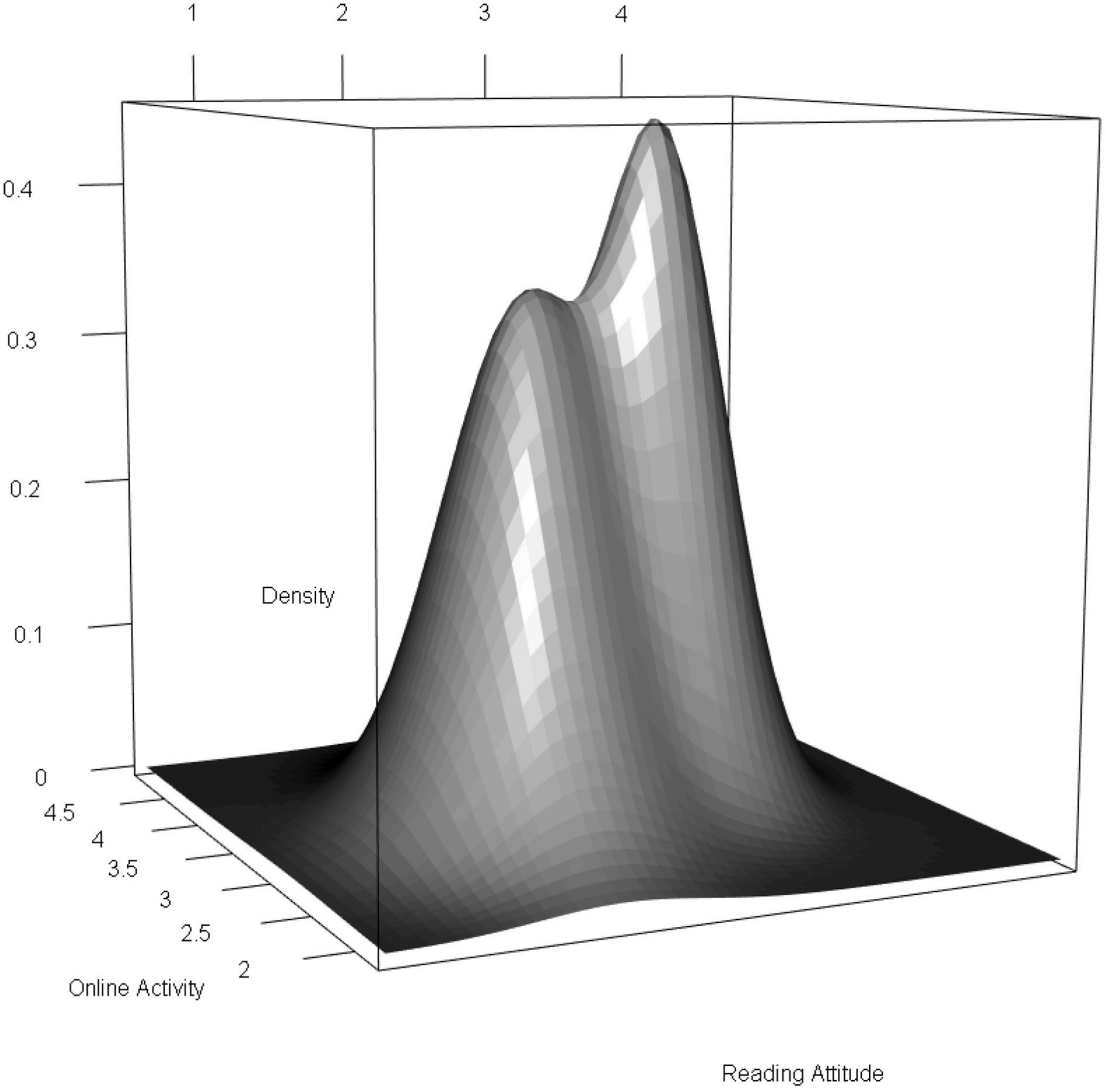
**Nonnormal bivariate distribution of the latent predictors**.

The nonnormal distribution of the latent predictor variables is illustrated in Figure 3. The nonnormality was mostly caused by reading attitudes (see class-specific means in Table 3).

#### Parameter estimates for model (c)

The results for Model (c), the multiple group model with (non)-linear effects, are presented in Table 4. We calculated two versions of standardized effects. The first version (${\hat{\theta }}^{•}$) refers to a within class standardization with class-specific means and variances^3^. The second version (${\hat{\theta }}^{{}^{\circ}}$) refers to pooled means and variances across groups (based on Equations 17 and 18). The results for female and male students were fairly similar for most parameters. The standardized regression coefficients were also fairly similar to those in Model (b) presented above. In both groups, the online activities had a negative quadratic effect on reading skills with standardized effect sizes of ${\hat{\gamma }}_{5,1}^{•}$ = −0.141 and ${\hat{\gamma }}_{5,2}^{•}=-0.111$ for female and male students, respectively, using the within class variances. The standardized quadratic effects of online activities based on the pooled variances only differed marginally from them with ${\hat{\gamma }}_{5,1}^{{}^{\circ}}=-0.181$ and ${\hat{\gamma }}_{5,2}^{{}^{\circ}}=-0.089$. The explained variances (based on the pooled variances) were fairly similar for female and male students with 42% (1−0.582) and 39% (1−0.606), respectively.

**Table 4:** **Unstandardized and standardized parameter estimates, standard errors, ***t***- and ***p***-values for the direct application of a nonlinear model with two observed classes (Gender)**.

	**$\hat{\theta }$**	***SE***	**${\hat{\theta }}^{•}$**	**${\hat{\theta }}^{{}^{\circ}}$**	***t***	***p***
*P*(*C* = 1)	0.500					
*P*(*C* = 2)	0.500					
***C* = 1 (FEMALE SUBJECTS)**						
γ_1, 1_	−0.037	0.110	0.527	0.474	−0.341	0.733
γ_2, 1_	0.721	0.240	0.116	0.146	3.004	0.003
γ_3, 1_	0.021	0.032	0.037	0.042	0.651	0.515
γ_4, 1_	0.020	0.015	0.049	0.047	1.346	0.178
γ_5, 1_	−0.108	0.039	−0.141	−0.181	−2.746	0.006
κ_1, 1_	2.875	0.031	0.000	0.266	92.081	< 0.001
κ_2, 1_	3.409	0.026	0.000	0.080	130.436	< 0.001
α_1_	−0.525	0.425	0.000	0.000	−1.236	0.217
ϕ_11, 1_	0.479	0.029	1.000	0.991	16.702	< 0.001
ϕ_21, 1_	0.055	0.019	0.157	0.327	2.914	0.004
ϕ_22, 1_	0.256	0.042	1.000	0.736	6.039	< 0.001
ψ_1_	0.025	0.003	0.647	0.582	9.858	< 0.001
***C* = 2 (MALE SUBJECTS)**						
γ_1, 2_	0.078	0.098	0.521	0.587	0.798	0.425
γ_2, 2_	0.429	0.122	0.183	0.145	3.525	< 0.001
γ_3, 2_	−0.008	0.024	−0.016	−0.016	−0.343	0.731
γ_4, 2_	0.023	0.018	0.047	0.054	1.241	0.215
γ_5, 2_	−0.053	0.020	−0.111	−0.089	−2.626	0.009
κ_1, 2_	2.505	0.031	0.000	−0.266	81.114	< 0.001
κ_2, 2_	3.315	0.032	0.000	−0.080	103.651	< 0.001
α_2_	−0.207	0.235	0.000	0.000	−0.880	0.379
ϕ_11, 2_	0.419	0.027	1.000	0.867	15.569	< 0.001
ϕ_21, 2_	0.135	0.021	0.316	0.803	6.442	< 0.001
ϕ_22, 2_	0.435	0.056	1.000	1.251	7.720	< 0.001
ψ_2_	0.026	0.003	0.606	0.606	9.192	< 0.001

*$\hat{\theta }$, unstandardized parameter estimates; SE, estimated standard errors; ${\hat{\theta }}^{•}$, standardized parameter estimates based on within class variances; ${\hat{\theta }}^{{}^{\circ}}$, standardized parameter estimates based on pooled variances; t, t-values based on standard errors; p, p-values for the t-values*.

The two groups differed mainly in the dispersion of online activities, where male students had a larger variability in their activities compared to female students (with ϕ_22, 1_ = 0.256 and ϕ_22, 2_ = 0.435) and in the average reading attitudes which were higher for females than for males (κ_1, 1_ = 2.875 vs. κ_1, 2_ = 2.505) with a standardized difference of $\kappa_{1,1}^{{}^{\circ}}-\kappa_{1,2}^{{}^{\circ}}=0.532$ based on the pooled means and variances. The standardization of the linear effects further showed that the standardized relationships were fairly similar across groups, despite the rather different unstandardized effects. By comparing the standardized effects, more meaningful differences can be seen because standardized effects refer to the effects of an average member of the respective group. In contrast, the unstandardized effects are complicated to compare, because the predictors' means in the two subgroups were different.

The interpretation of the two standardizations based on either within class parameters or pooled parameters refer to two different frames of reference. For example, the linear effect of reading attitude on reading skills, $\gamma_{1,1}^{•}=0.527$, based on the within class means and variances is the standardized effect for female students with average reading attitude in this group (i.e., κ_1, 1_ = 2.875). This standardized parameter can be used to compare the effect to other studies in which effects for female students are examined. The standardized effect, $\gamma_{1,1}^{{}^{\circ}}=0.474$, based on the pooled means and variances refers to female subjects with average reading skills referring to the complete sample (i.e., κ_1_ = 0.5κ_1, 1_+0.5κ_1, 2_ = 2.690). This effect can be used to meaningfully compare the groups within this study: for subjects with the same average reading skills (of κ_1_ = 2.690) the standardized effect is 0.474 for female and 0.587 for male students.

**Figure 4:**
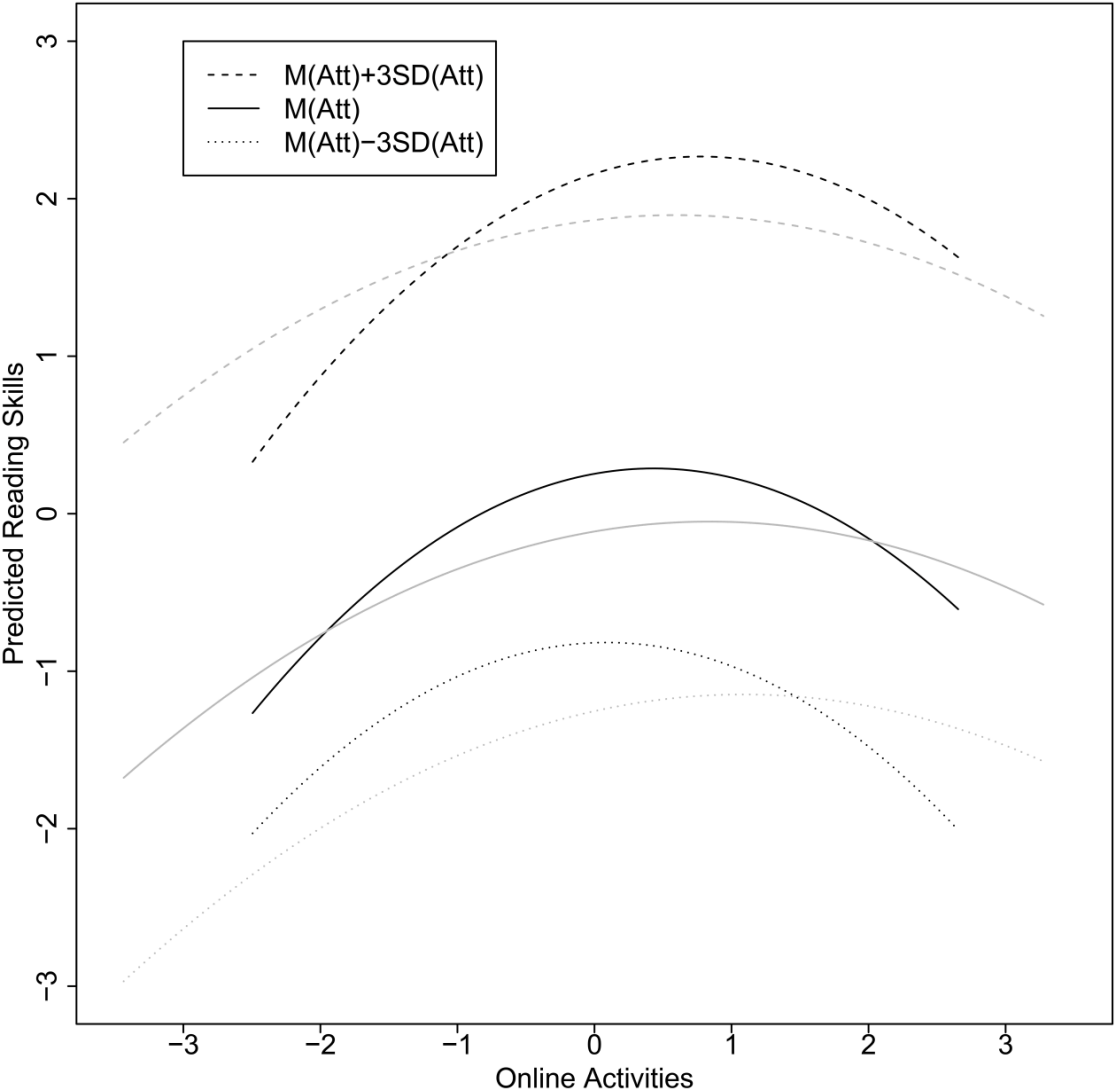
**Simple slopes for female (black lines) and male students (gray lines) based on a standardization using the pooled variances**. The relationship for online activities and reading skills were estimated for low, average, and high reading attitudes.

Figure 4 illustrates the relationships between the variables using simple slopes based on standardized effects using the pooled means and variances.

The standardization of the linear and nonlinear regression coefficients in this example allows us to compare the effect sizes between different models and subgroups. Furthermore, it allows researchers to compare the results here to other samples where, for example, the means of the latent variables are different and therefore the unstandardized coefficients could not be meaningfully compared. In addition, the percentage of variance explained for the latent criterion could be calculated ($1-\psi_{g}^{•}$), which could not have been done in a straightforward manner without the derivation of the model implied variance of η_*g*_.

## Discussion

In this article, we provided a standardization procedure for linear and nonlinear SEM including traditional parametric nonlinear SEM (e.g., LMS; Klein and Moosbrugger, 2000), semiparametric SEMM (e.g., Jedidi et al., 1997b), and recently proposed mixture approaches for nonnormal variables (e.g., NSEMM; Kelava and Nagengast, 2012; Kelava et al., 2014).

The formulas provided are more general than those provided by Wen et al. (2010) because they do not need the strong assumption of centered and normally distributed latent predictor variables. Although the formulas provided here refer to Equations (4) and (16) with a single dependent variable and two predictor variables, an extension to more variables is straightforward and can be inferred directly from the formulas. These formulas are useful in at least three situations.

First, they are useful when the product indicator approaches are not used and a standardization of a nonlinear model is needed. For example, this is the case when popular commercial latent variable modeling software like Mplus (Muthén and Muthén, 1998–2012) is applied to examine nonlinear interaction or quadratic effects (with the XWITH command). Since estimates for the variances of the latent product terms (e.g., ξ_1_ξ_2_) are not needed, the proposed standardization procedure can be applied to all approaches for the estimation of nonlinear interaction and quadratic effects (e.g., the LMS approach Klein and Moosbrugger, 2000, the QML approach; Klein and Muthén, 2007, or the NSEMM approach Kelava et al., 2014).

Second, the formulas are also useful when standardizing linear as well as nonlinear effects in cases when centering the observed variables does not imply centered latent variables (e.g., in latent mixture models or in multiple group SEM). In these situations, the class- or group-specific means contain information about the dissimilarity of the latent subpopulations that are extracted or are specified in the case of observed groups. This aspect needs to be taken into account during the standardization procedure. In the presence of nonlinearity, the linear effects are not invariant against a transformation of data (Moosbrugger et al., 1997). The standardization as proposed here allows researchers to interpret the effect size of a linear effect for subjects with an average value in the respective predictor variable. Researchers have previously proposed centering variables in interaction models so that an interpretation of the linear effects is meaningful (i.e., the sample mean in the respective predictor variable). In the case of latent mixture models or in multiple group SEM, centering the observed variables does not lead to centered predictor variables within each group and hence, centering is not meaningful in these scenarios. The standardization procedure presented in this paper allows researchers to refer to effects of centered variables. Thus, results from multiple group/class models can be compared to results from single group models with centered variables.

Third, the formulas are additionally useful for standardization in cases when the variables are nonnormally distributed. Several simulation studies have shown that nonnormality of the variables may introduce a bias in the parameter estimation of the nonlinear effects (Marsh et al., 2004; Cham et al., 2012; Kelava and Nagengast, 2012; Brandt et al., 2014; Kelava et al., 2014) particularly for distribution analytic approaches (e.g., LMS or QML). This bias is due to a misspecified mean and covariance structure when data are nonnormal because the estimates are derived under the assumption of normality of the latent predictor variables and measurement error variables. Hence, considering nonnormal latent distributions (e.g., by using mixture distributions) is essential for an unbiased estimation of nonlinear effects. In this article, we showed that it is again necessary to take the nonnormality of the variables into account for a correct standardization.

In practical applications of multiple group models, it is sometimes desirable to standardize effects with a pooled variance estimate or the variance of a reference group instead of within class variances. The procedure proposed here allows for all of these cases by using the respective variances derived for the direct and the indirect application of mixture models. We illustrated this with an empirical example from education science. It is obvious that the standardization depends on a reliable estimate for the class-specific variances and therefore classes (or observed groups) should not be too small.

From a substantive user's perspective, the formulas can be applied by simple algebra after parameter estimates were obtained. The standardization procedure is implemented in the package nlsem for the R system for statistical computing (R Core Team, 2015), which is freely available at http://CRAN.r-project.org/package=nlsem. The package can also be used to standardize effects estimated with Mplus.

Besides the desirable properties of the proposed standardization procedure, it is important to note that there are still situations of nonlinear SEM that are not covered by this procedure. For example, semiparametric spline models for latent variables (Yang and Dunson, 2010; Kelava and Brandt, 2014) require separate procedures that account for the specific structure of these regression functions. Furthermore, the proposed procedure can be extended for multilevel data structures. In cases where multilevel nonlinear structural equation models are given (e.g., Leite and Zuo, 2011; Nagengast et al., 2013; Kelava and Brandt, 2014), adapted procedures are needed that still offer interpretable results. Future work is needed to address these remaining issues.

### Conflict of interest statement

The authors declare that the research was conducted in the absence of any commercial or financial relationships that could be construed as a potential conflict of interest.

## Footnotes

^1^A latent multilevel model that included a latent random intercept variable for reading skills did not substantively change the parameter estimates presented here.

^2^Reading skills: average of items R06, R102, R219, R220, R414, R447, R452, and R458. Attitude toward reading: items ST24Q01–ST24Q11. Online activities: items ST26Q01–ST26Q07.

^3^For a visualization of standardized group differences in an empirical data set see Figure 4.
