# Supplementary material for: The Standardization of Linear and Nonlinear Effects in Direct and Indirect Applications of Structural Equation Mixture Models for Normal and Nonnormal Data
Source: Front Psychol. 2015 Nov 30;6:1813. doi: 10.3389/fpsyg.2015.01813 (PMC4663265; doi:10.3389/fpsyg.2015.01813)
Supplement: Supplementary file 1 [file DataSheet1.PDF]

The Standardization of Linear and Nonlinear Effects in Direct and Indirect Applications of Structural Equation Mixture Models for Normal and Nonnormal Data

Holger Brandt*, Nora Umbach, and Augustin Kelava
Hector Research Institute of Education Sciences and Psychology
Eberhard Karls Universität Tübingen

Supplementary Material A: Derivation of the Model Implied Variance of η

In this section, we derive the model implied variance of the latent dependent variable η. For this derivation, the variances and covariances of the latent predictors and the latent product terms are required. Such variances and covariances can be specified for given random variables x_i, x_j, x_k, x_l (Bohrnstedt & Goldberger, 1969) by

$$V(x_i x_j) = \mu_i^2 \nu_{jj} + \mu_j^2 \nu_{ii} + 2\mu_i\mu_j\nu_{ij} - \nu_{ij}^2 + 2\mu_i\nu_{ijj} + 2\mu_j\nu_{iij} + \nu_{iijj} \tag{A1}$$

$$\begin{aligned} C(x_i x_j, x_k x_l) = {} & \mu_i\mu_k\nu_{jl} + \mu_i\mu_l\nu_{jk} + \mu_j\mu_k\nu_{il} + \mu_j\mu_l\nu_{ik} - \nu_{ij}\nu_{kl} \\ & + \mu_i\nu_{jkl} + \mu_j\nu_{ikl} + \mu_k\nu_{ijl} + \mu_l\nu_{ijk} + \nu_{ijkl} \end{aligned} \tag{A2}$$

$$C(x_i x_j, x_l) = \mu_i\nu_{jl} + \mu_j\nu_{il} + \nu_{ijl}, \tag{A3}$$

where $\mu_{\cdot}$ is the first moment and $\nu_{\cdot\cdot}$, $\nu_{\cdot\cdot\cdot}$, and $\nu_{\cdot\cdot\cdot\cdot}$ are the second to fourth central moments of the respective variables. If variables are normally distributed, the third central moments are zero and the fourth central moments are a simple function of the second central moments. In this case, the calculation of the variances and covariances is straightforward and is derived for the model specified in Equation (4) for the class-specific standardization (see below), where normality of the variables can be assumed because the latent classes are extracted explicitly under this assumption.

If variables are nonnormally distributed, the calculation of the third and fourth central moments needs a more complex specification. If a mixture of normal distributions is used to approximate the nonnormal variables' distribution, the moments can be derived analytically as follows:

1. Calculate the noncentral class-specific moments based on the assumption of normally distributed variables within each latent class.

*Email: holger.brandt@uni-tuebingen.de

2. Calculate the noncentral moments of the nonnormally distributed mixture variable.

3. Using these noncentral moments, calculate the central moments of the nonnormally distributed mixture variable.

1. Noncentral class-specific moments. The second to fourth class-specific noncentral moments $\mu_{..}$, $\mu_{...}$, and $\mu_{....}$ for variables $x_{i,g}, x_{j,g}, x_{k,g}, x_{l,g}$ are given in general as

$$\mu_{ij,g} = \mu_{i,g}\mu_{j,g} + \nu_{ij,g} \tag{A4}$$
$$\mu_{ijk,g} = \nu_{ijk,g} + \mu_{ij,g}\mu_{k,g} + \mu_{ik,g}\mu_{j,g} + \mu_{jk,g}\mu_{i,g} - 2\mu_{i,g}\mu_{j,g}\mu_{k,g} \tag{A5}$$
$$\begin{aligned}\mu_{ijkl,g} = {} & \nu_{ijkl,g} + \mu_{ijk,g}\mu_{l,g} + \mu_{ijl,g}\mu_{k,g} + \mu_{ikl,g}\mu_{j,g} + \mu_{jkl,g}\mu_{i,g} - \mu_{ij,g}\mu_{k,g}\mu_{l,g} - \mu_{ik,g}\mu_{j,g}\mu_{l,g} \\ & - \mu_{il,g}\mu_{j,g}\mu_{k,g} - \mu_{jk,g}\mu_{i,g}\mu_{l,g} - \mu_{jl,g}\mu_{i,g}\mu_{k,g} - \mu_{kl,g}\mu_{i,g}\mu_{j,g} + 3\mu_{i,g}\mu_{j,g}\mu_{k,g}\mu_{l,g}.\end{aligned} \tag{A6}$$

Under the assumption that the variables are normally distributed within each mixture component $g = 1, \ldots, G$ the central class-specific third and fourth moments are specified by

$$\nu_{ijk,g} = 0 \tag{A7}$$
$$\nu_{ijkl,g} = \nu_{ij,g}\nu_{kl,g} + \nu_{ik,g}\nu_{jl,g} + \nu_{il,g}\nu_{jk,g}. \tag{A8}$$

As a consequence, the noncentral class-specific moments can be calculated using the class-specific means and (co-)variances of the variables.

2. Noncentral moments of mixture variables. In general, the k-th noncentral moment of a mixture variable is a weighted sum of the k-th noncentral class-specific moments (Haas, Mittnik, & Paolella, 2009):

$$\mu^{(k)} = \sum_g \pi_g \mu_g^{(k)}. \tag{A9}$$

3. Central moments of mixture variables. Analogous to Equations (A4) to (A6), the central moments of the mixture variables are then given by

$$\nu_{ij} = \mu_{ij} - \mu_i\mu_j \tag{A10}$$
$$\nu_{ijk} = \mu_{ijk} - \mu_{ij}\mu_k - \mu_{ik}\mu_j - \mu_{jk}\mu_i + 2\mu_i\mu_j\mu_k \tag{A11}$$
$$\begin{aligned}\nu_{ijkl} = {} & \mu_{ijkl} - \mu_{ijk}\mu_l - \mu_{ijl}\mu_k - \mu_{ikl}\mu_j - \mu_{jkl}\mu_i + \mu_{ij}\mu_k\mu_l + \mu_{ik}\mu_j\mu_l \\ & + \mu_{il}\mu_j\mu_k + \mu_{jk}\mu_i\mu_l + \mu_{jl}\mu_i\mu_k + \mu_{kl}\mu_i\mu_j - 3\mu_i\mu_j\mu_k\mu_l,\end{aligned} \tag{A12}$$

with the (noncentral) moments $\mu_{.}$, $\mu_{..}$, $\mu_{...}$ and $\mu_{....}$ obtained from Equation (A9).

In the next two subsections, we use these general formulas to derive the model implied (co-)variances first for the direct and then for the indirect application of a mixture model with nonlinear effects as specified in Equations (4) and (16).

Standardization of the Direct Application (Class-Specific Standardization)

Concerning the class-specific standardization in the direct application, the model implied class-specific variance of η_g for a model that is given in Equation (4) is specified by

$$Var(\eta_g) = \phi_{00g} = \boldsymbol{\gamma}_g \boldsymbol{\Phi}^*_g \boldsymbol{\gamma}'_g + \psi_g \tag{A13}$$

with parameter vector $\boldsymbol{\gamma}_g = (\gamma_{1,g}, \ldots, \gamma_{5,g})'$ and 5×5 class-specific covariance matrix $Cov\left((\xi_{1,g}, \xi_{2,g}, \xi_{1,g}\xi_{2,g}, \xi^2_{1,g}, \xi^2_{2,g})'\right) = \boldsymbol{\Phi}^*_g$ for the latent predictor variables and product terms. The nonredundant elements of $\boldsymbol{\Phi}^*_g$ are specified using Equations (A1) to (A3) and moments specified in Equations (A4) to (A8), and using the notation $\mu_{\cdot,g} = \kappa_{\cdot,g}$ and $\nu_{\cdot\cdot,g} = \phi_{\cdot\cdot,g}$:

$$\begin{aligned}
\phi_{13,g} &= \kappa_{1,g}\phi_{12,g} + \kappa_{2,g}\phi_{11,g} \\
\phi_{23,g} &= \kappa_{1,g}\phi_{22,g} + \kappa_{2,g}\phi_{12,g} \\
\phi_{33,g} &= \kappa^2_{1,g}\phi_{22,g} + \kappa^2_{2,g}\phi_{11,g} + 2\kappa_{1,g}\kappa_{2,g}\phi_{12,g} + \phi_{11,g}\phi_{22,g} + \phi^2_{12,g} \\
\phi_{14,g} &= 2\kappa_{1,g}\phi_{11,g} \\
\phi_{24,g} &= 2\kappa_{1,g}\phi_{12,g} \\
\phi_{34,g} &= 2\kappa^2_{1,g}\phi_{12,g} + 2\kappa_{1,g}\kappa_{2,g}\phi_{11,g} + 2\phi_{11,g}\phi_{12,g} \\
\phi_{44,g} &= 4\kappa^2_{1,g}\phi_{11,g} + 2\phi^2_{11,g} \\
\phi_{15,g} &= 2\kappa_{2,g}\phi_{12,g} \\
\phi_{25,g} &= 2\kappa_{2,g}\phi_{22,g} \\
\phi_{35,g} &= 2\kappa^2_{2,g}\phi_{12,g} + 2\kappa_{1,g}\kappa_{2,g}\phi_{22,g} + 2\phi_{22,g}\phi_{12,g} \\
\phi_{45,g} &= 4\kappa_{1,g}\kappa_{2,g}\phi_{12,g} + 2\phi^2_{12,g} \\
\phi_{55,g} &= 4\kappa^2_{2,g}\phi_{22,g} + 2\phi^2_{22,g},
\end{aligned}$$

since

$$\begin{aligned}
\nu_{1111,g} &= 3\phi^2_{11,g} \\
\nu_{1112,g} &= 3\phi_{11,g}\phi_{12,g} \\
\nu_{1122,g} &= \phi_{11,g}\phi_{22,g} + 2\phi^2_{12,g} \\
\nu_{1222,g} &= 3\phi_{22,g}\phi_{12,g} \\
\nu_{2222,g} &= 3\phi^2_{22,g}.
\end{aligned}$$

Standardization of the Indirect Application

For the indirect application, a global standardization is based on the covariance matrix of the mixture variables. For a model such as one specified in Equation (16), the model implied variance of η is given by

$$Var(\eta) = \phi_{00} = \boldsymbol{\gamma}\boldsymbol{\Phi}^*\boldsymbol{\gamma}' + \psi \tag{A14}$$

with $\boldsymbol{\gamma} = (\gamma_1, \ldots, \gamma_5)'$ and 5×5 covariance matrix $\mathbf{\Phi}^*$ for the latent predictor variables and product terms. The (co-)variances $(\phi_{11}, \phi_{12}, \phi_{22})$ and means (κ_1, κ_2) of the latent predictors are specified in Equation (17) and (18). The remaining nonredundant elements of $\mathbf{\Phi}^*$ are given by:

$$
\begin{aligned}
\phi_{13} &= \kappa_1\phi_{12} + \kappa_2\phi_{11} + \nu_{112} \\
\phi_{23} &= \kappa_1\phi_{22g} + \kappa_2\phi_{12} + \nu_{122} \\
\phi_{33} &= \kappa_1^2\phi_{22} + \kappa_2^2\phi_{11} + 2\kappa_1\kappa_2\phi_{12} - \phi_{12}^2 + 2\kappa_1\nu_{122} + 2\kappa_2\nu_{112} + \nu_{1122} \\
\phi_{14} &= 2\kappa_1\phi_{11} + \nu_{111} \\
\phi_{24} &= 2\kappa_1\phi_{12} + \nu_{112} \\
\phi_{34} &= 2\kappa_1^2\phi_{12} + 2\kappa_1\kappa_2\phi_{11} - \phi_{11}\phi_{12} + 3\kappa_1\nu_{112} + \kappa_2\nu_{111} + \nu_{1112} \\
\phi_{44} &= 4\kappa_1^2\phi_{11} - \phi_{11}^2 + 4\kappa_1\nu_{111} + \nu_{1111} \\
\phi_{15} &= 2\kappa_2\phi_{12} + \nu_{122} \\
\phi_{25} &= 2\kappa_2\phi_{22} + \nu_{222} \\
\phi_{35} &= 2\kappa_2^2\phi_{12} + 2\kappa_1\kappa_2\phi_{22} - \phi_{22}\phi_{12} + 3\kappa_2\nu_{122} + \kappa_1\nu_{222} + \nu_{1222} \\
\phi_{45} &= 4\kappa_1\kappa_2\phi_{12} - \phi_{11}\phi_{22} + 2\kappa_1\nu_{122} + 2\kappa_2\nu_{112} + \nu_{1122} \\
\phi_{55} &= 4\kappa_2^2\phi_{22} - \phi_{22}^2 + 4\kappa_2\nu_{222} + \nu_{2222}.
\end{aligned}
$$

The derivation of the third and fourth central moments is conducted with the steps described above.

1. The third and fourth noncentral class-specific moments (cf. Equations (A5) to (A8)) are given by

$$
\begin{aligned}
\mu_{111,g} &= \kappa_{1,g}^3 + 3\kappa_{1,g}\phi_{11,g} \\
\mu_{112,g} &= \kappa_{1,g}^2\kappa_{2,g} + \kappa_{2,g}\phi_{11,g} + 2\kappa_{1,g}\phi_{12,g} \\
\mu_{122,g} &= \kappa_{2,g}^2\kappa_{1,g} + \kappa_{1,g}\phi_{22,g} + 2\kappa_{2,g}\phi_{12,g} \\
\mu_{222,g} &= \kappa_{2,g}^3 + 3\kappa_{2,g}\phi_{22,g}
\end{aligned}
$$

and

$$
\begin{aligned}
\mu_{1111,g} &= 3\phi_{11,g}^2 + \kappa_{1,g}^4 + 6\kappa_{1,g}^2\phi_{11,g} \\
\mu_{1112,g} &= 3\phi_{11,g}\phi_{12,g} + \kappa_{1,g}^3\kappa_{2,g} + 3\kappa_{1,g}\kappa_{2,g}\phi_{11,g} + 3\kappa_{1,g}^2\phi_{12,g} \\
\mu_{1122,g} &= \phi_{11,g}\phi_{22,g} + 2\phi_{12,g}^2 + \kappa_{1,g}^2\kappa_{2,g}^2 + \kappa_{2,g}^2\phi_{11,g} + 4\kappa_{2,g}\kappa_{1,g}\phi_{12,g} + \kappa_{1,g}^2\phi_{22,g} \\
\mu_{1222,g} &= 3\phi_{22,g}\phi_{12,g} + \kappa_{2,g}^3\kappa_{1,g} + 3\kappa_{1,g}\kappa_{2,g}\phi_{22,g} + 3\kappa_{2,g}^2\phi_{12,g} \\
\mu_{2222,g} &= 3\phi_{22,g}^2 + \kappa_{2,g}^4 + 6\kappa_{2,g}^2\phi_{22,g},
\end{aligned}
$$

respectively, with class-specific means $(\kappa_{1,g}, \kappa_{2,g})$ and (co-)variances $(\phi_{11,g}, \phi_{22,g}, \phi_{12,g})$.

2. The noncentral moments of the mixtures can be calculated as the weighted sum of the class-specific moments using Equation (A9).

3. The central moments of the mixture variables are then given by (cf. Equations (A11) to (A12))

$$\begin{aligned}
\nu_{111} &= \mu_{111} - \kappa_1^3 - 3\kappa_1\phi_{11} \\
\nu_{112} &= \mu_{112} - \kappa_1^2\kappa_2 - \kappa_2\phi_{11} - 2\kappa_1\phi_{12} \\
\nu_{122} &= \mu_{122} - \kappa_2^2\kappa_1 - \kappa_1\phi_{22} - 2\kappa_2\phi_{12} \\
\nu_{222} &= \mu_{222} - \kappa_2^3 - 3\kappa_2\phi_{22}
\end{aligned}$$

and

$$\begin{aligned}
\nu_{1111} &= \mu_{1111} - 4\mu_{111}\kappa_1 + 3\kappa_1^4 + 6\kappa_1^2\phi_{11} \\
\nu_{1112} &= \mu_{1112} - 3\mu_{112}\kappa_1 + 3\kappa_1^3\kappa_2 + 3\kappa_1\kappa_2\phi_{11} + 3\kappa_1^2\phi_{12} - \mu_{111}\kappa_2 \\
\nu_{1122} &= \mu_{1122} - 2\mu_{112}\kappa_2 - 2\mu_{122}\kappa_1 + 3\kappa_1^2\kappa_2^2 + \kappa_2^2\phi_{11} + 4\kappa_1\kappa_2\phi_{12} + \kappa_1^2\phi_{22} \\
\nu_{1222} &= \mu_{1222} - 3\mu_{122}\kappa_2 + 3\kappa_2^3\kappa_1 + 3\kappa_1\kappa_2\phi_{22} + 3\kappa_2^2\phi_{12} - \mu_{222}\kappa_1 \\
\nu_{2222} &= \mu_{2222} - 4\mu_{222}\kappa_2 + 3\kappa_2^4 + 6\kappa_2^2\phi_{22},
\end{aligned}$$

respectively.

Supplementary Material B: Illustration of a Standardization with Group-Specific Variances and Pooled Variances

Table B1

Fictitious example for the standardized relationship between shoe size (shoe) and body height (height) for male and female subjects ($g = 1, 2$) in two studies ($s = 1, 2$).

	Study 1 ($S = 1$)		Study 2 ($S = 2$)	
	Male ($G = 1$)	Female ($G = 2$)	Male ($G = 1$)	Female ($G = 2$)
$P(G = g)$	0.75	0.25	0.25	0.75
$E(shoe \mid G = g, S = s)$	9.00	7.00	42.51	40.11
$V(shoe \mid G = g, S = s)$	0.50	0.45	0.72	0.65
$E(height \mid G = g, S = s)$	69.41	63.86	1.76	1.62
$V(height \mid G = g, S = s)$	20.07	19.27	0.01	0.01
$E(shoe \mid S = s)$	8.50	8.50	40.71	40.71
$V(shoe \mid S = s)$	1.24	1.24	1.75	1.75
$E(height \mid S = s)$	68.02	68.02	1.66	1.66
$V(height \mid S = s)$	25.65	25.65	0.02	0.02
β	3.17	3.93	0.07	0.08
$\beta^\bullet$	0.50	0.60	0.50	0.60
β°	0.70	0.86	0.69	0.86

Note. $E(\cdot)$ – expected value; $V(\cdot)$ – variance; β – unstandardized regression coefficient; $\beta^\bullet$ – standardized regression coefficient based on within class variances; β° – standardized regression coefficient based on pooled variances.

The following fictitious example concerning the relationship between shoe size and body height illustrates the differences between a within class standardization and a standardization based on pooled variances. Table B1 includes (fictitious) means and variances for male and female subjects in two different studies. In study 1, the mean shoe size for men and women were $E(shoe|G = 1, S = 1) = 9$ and $E(shoe|G = 2, S = 1) = 7$ (American sizes) with (plausible) variances of $V(shoe|G = 1, S = 1) = .50$ and $V(shoe|G = 2, S = 1) = .45$. In study 2, the same information concerning the means and variances were used, but they were transformed to the German shoe size system (German shoe size $\approx 31.71 + 1.20 \cdot$ American shoe size). The average body heights in study 1 were assumed to be $E(height|G = 1, S = 1) = 69.41$ and $E(height|G = 2, S = 1) = 63.86$ inches for men and women, respectively, with variances of $V(height|G = 1, S = 1) = 20.07$ and $V(height|G = 2, S = 1) = 19.27$ (McDowell, Fryar, Ogden, & Flegal, 2008). Again, the same information about the means and variances held in study 2, but were transformed from inches to the metric system. Finally, we assumed that reasonable correlations between shoe size and body height were .5 for men and .6 for women. The only substantive difference between the two studies was that in study 1 75% of the subjects were male and in study 2 only 25% were male.

Based on these assumptions, the sample means $(E(shoe|S = s), E(height|S = s))$ and variances $(V(shoe|S = s), V(height|S = s))$ for each study were calculated as well

as the unstandardized regression coefficients (β), and finally, the standardized regression coefficients based on the within variances ($\beta^{\bullet}$)[1] and the pooled variances (β°)[2].

The unstandardized regression coefficients were different across groups and different across studies. The within group standardized regression coefficients were the same in the respective groups of both studies ($\beta^{\bullet} = .5$ for male and $\beta^{\bullet} = .6$ for female subjects). The standardized regression coefficients can be meaningfully used to compare effects across studies, that is, it is meaningful to compare, for example, male subjects across studies: For an increase of 1 SD in shoe size ($SD(shoe|G = 1, S = 1) = \sqrt{.50} = .71$ in study 1 and $SD(shoe|G = 1, S = 2) = \sqrt{.72} = .85$ in study 2) the body height increases by .50 SD (which is $SD(height|G = 1, S = 1) = \sqrt{20.07} = 4.48$ feet in study 1 and $SD(height|G = 1, S = 2) = \sqrt{.01} = .1$ meters in study 2). The same logic applies for female subjects ($G = 2$). The standardization based on the within group variances can also be used to compare different groups within one study; however, it needs to be considered that the information about differences in variances are not taken into account.

The pooled means and variances depend on the proportions of the two groups. They can be used, for example, to compare different subgroups within a study in a unified reference system. For example, in study 1, an increase of 1 SD (based on the pooled variance $SD(shoe|S = 1) = \sqrt{1.24} = 1.11$) in shoe size leads to an increase in body height of .70 SD for male and of .86 SD for female subjects with an $SD(height|S = 1) = \sqrt{25.65} = 5.06$ for the body height in both groups. Note that due to the common metric – the pooled variance – the effect sizes are directly comparable across groups.

The standardization based on the pooled variances also allows one to compare other parameters based on a common metric. For example, the standardized means based on a within standardization are $E^{\bullet}(shoe|G = 1, S = s) = E^{\bullet}(shoe|G = 2, S = s) = 0$ for both groups and hence differences between the two groups are lost. The standardized means based on the pooled parameters keep information about the differences in the groups intact because while the pooled standardized mean is zero ($E^{\circ}(shoe|S = s) = 0$), the group-specific standardized means are z-values of the form $E^{\circ}(shoe|G = g, S = s) = [E(shoe|G = g, S = s) - E(shoe|S = s)]/SD(shoe|S = s)$ (which are $E^{\circ}(shoe|G = 1, S = 1) = .44$ for male and $E^{\circ}(shoe|G = 2, S = 1) = -1.35$ for female subjects in study 1).[3]

References

Bohrnstedt, G. W., & Goldberger, A. S. (1969). On the exact covariance of products of random variables. *American Statistical Association Journal*, *64*, 1439–1442. doi: 10.1080/01621459.1969.10501069

Haas, M., Mittnik, S., & Paolella, M. (2009). Asymmetric multivariate normal mixture garch. *Computational Statistics & Data Analysis*, *53*, 2129–2154. doi: 10.1016/j.csda.2007.12.018

McDowell, M. A., Fryar, C. D., Ogden, C. L., & Flegal, K. M. (2008). Anthropometric reference data for children and adults: United states, 2003-2006. *National Health Statistics Report*, *10*, 1–45. Retrieved from `http://www.cdc.gov/nchs/data/nhsr/nhsr010.pdf`

[1] $\beta^{\bullet} = \beta \cdot SD(shoe|G = g, S = s)/SD(height|G = g, S = s)$

[2] $\beta^{\circ} = \beta \cdot SD(shoe|S = s)/SD(height|S = s)$

[3] For a visualization of standardized differences in an empirical data set see Figure 4.
